# Clinical Manifestations Associated with Neurocysticercosis: A Systematic Review

**DOI:** 10.1371/journal.pntd.0001152

**Published:** 2011-05-24

**Authors:** Hélène Carabin, Patrick Cyaga Ndimubanzi, Christine M. Budke, Hai Nguyen, Yingjun Qian, Linda Demetry Cowan, Julie Ann Stoner, Elizabeth Rainwater, Mary Dickey

**Affiliations:** 1 Department of Biostatistics and Epidemiology, University of Oklahoma Health Sciences Center, Oklahoma City, Oklahoma, United States of America; 2 Department of Veterinary Integrative Biosciences, College of Veterinary Medicine and Biomedical Sciences, Texas A&M University, College Station, Texas, United States of America; 3 National Institute of Parasitic Diseases, Shanghai, People's Republic of China; 4 Department of Medicine, University of Oklahoma Health Sciences Center, Oklahoma City, Oklahoma, United States of America; 5 Department of Health Promotion Sciences, University of Oklahoma Health Sciences Center, Oklahoma City, Oklahoma, United States of America; Universidad Nacional Autónoma de México, Mexico

## Abstract

**Background:**

The clinical manifestations of neurocysticercosis (NCC) are poorly understood. This systematic review aims to estimate the frequencies of different manifestations, complications and disabilities associated with NCC.

**Methods:**

A systematic search of the literature published from January 1, 1990, to June 1, 2008, in 24 different electronic databases and 8 languages was conducted. Meta-analyses were conducted when appropriate.

**Results:**

A total of 1569 documents were identified, and 21 included in the analysis. Among patients seen in neurology clinics, seizures/epilepsy were the most common manifestations (78.8%, 95%CI: 65.1%–89.7%) followed by headaches (37.9%, 95%CI: 23.3%–53.7%), focal deficits (16.0%, 95%CI: 9.7%–23.6%) and signs of increased intracranial pressure (11.7%, 95%CI: 6.0%–18.9%). All other manifestations occurred in less than 10% of symptomatic NCC patients. Only four studies reported on the mortality rate of NCC.

**Conclusions:**

NCC is a pleomorphic disease linked to a range of manifestations. Although definitions of manifestations were very rarely provided, and varied from study to study, the proportion of NCC cases with seizures/epilepsy and the proportion of headaches were consistent across studies. These estimates are only applicable to patients who are ill enough to seek care in neurology clinics and likely over estimate the frequency of manifestations among all NCC cases.

## Author Summary

Neurocysticercosis is an infection of the brain with the flatworm *Taenia solium* which is normally transmitted between humans and pigs. Sometimes, humans can infect other humans and the larva of the parasite can go the brain, causing the disease neurocysticercosis. There has never been a systematic review of what clinical signs are found among people with neurocysticercosis. We conducted a thorough review of the literature to answer this question. We reviewed 1569 and 21 were of a sufficient quality to be included in the final analysis. Among neurocysticercosis patients who are seeking care in neurology clinics, about 79% have seizures/epilepsy, 38% severe headaches, 16% focal deficits and 12% signs of increased intracranial pressure. Several other symptoms were also reported in less than 10% of patients. People with neurocysticercosis who seek care in neurology clinics show a whole range of manifestations. Clinicians should be encouraged to consider neurocysticercosis in their differential diagnosis when a patient presented with one of the symptoms described in this review. This would ultimately improve the estimates of the frequency of symptoms associated with neurocysticercosis.

## Introduction

Neurocysticercosis (NCC) is primarily found in countries with poor sanitation and hygiene and improper slaughterhouse services. However, due to globalization and immigration, NCC is increasingly being reported in developed countries [1]. Humans become infected by ingesting *Taenia solium* eggs that later develop into oncospheres. These larvae can migrate to any organ in the body, but most reports have focused on cysts located in the Central Nervous System (CNS), eyes, muscles or subcutaneous tissues. The larvae have been found in several locations in the CNS. This diversity of locations is believed to partly explain the range of NCC's clinical manifestations. In addition, the signs and symptoms associated with NCC depend on the larvae's number, developmental stage (active, transitional or calcified), on the duration of the infection and the host's immune response [2].

Seizures and epilepsy are considered to be the most common manifestations of NCC. However, several other neurological disorders can also occur [3]. Unfortunately, these less common manifestations are rarely recognized as being linked to NCC, especially in low resource countries where imaging technology is scarce [4]. Thus, data on the full range of clinical expression of NCC are lacking, although such data are essential to accurately estimate the burden of NCC on different communities. This systematic review aims to estimate the frequency of the main clinical manifestations associated with NCC.

## Methods

A systematic search of the literature, including documents published from January 1, 1990 to June 1, 2008, was conducted to capture data on clinical manifestations associated with NCC.

### Search strategy and data source

PubMed, Commonwealth Agricultural Bureau (CAB) Abstracts, and 23 international databases were searched for data on NCC manifestations. Articles published in Chinese, English, French, Portuguese, Spanish, Italian, Romanian and German were searched. Two different searches were launched to cover both clinical manifestations and mortality associated with NCC infection. For the clinical manifestations, our search strategy in PubMed included terms: "Cysticercosis/complications" [MeSH] OR "Cysticercosis/history" [MeSH] OR “Cysticercosis/pathology" [MeSH] OR "Cysticercosis/psychology" [MeSH] OR "Cysticercosis/radiography" [MeSH] OR "Cysticercosis/radionuclide imaging" [MeSH] OR "Cysticercosis/ultrasonography" [MeSH]. CAB Abstracts and the international search engines were queried using the following keywords: “*Taenia solium*”, “taeniasis” or “taeniosis”, “cysticercosis”, and “neurocysticercosis”. One Thesis in Medicine from Burkina Faso was identified through contacts in Sub-Saharan Africa and was included.

For mortality associated with NCC, PubMed was searched using the terms: “cysticercosis/mortality” [MeSH] OR "neurocysticercosis/mortality" [MeSH]. In CAB Abstracts and the international search engines the keywords “neurocysticercosis and mortality” were used.

### Inclusion and exclusion criteria

Documents reporting valid (defined as an absence of major biases, see later), original data on clinical manifestations associated with NCC were eligible for inclusion. Books and conference abstracts were excluded because they were unlikely to have sufficient details on the methodology used.

All documents retrieved were screened based on the title and the abstract. The exclusion criteria for phase I were: 1) wrong agent; 2) animal data only; 3) no original data on the frequency of NCC's clinical manifestations; 4) case series with less than 20 participants; 5) review article without original data; and 6) editorials or letters to the editors without original data. Documents without abstracts were included in the next phase. After phase I, all eligible full text documents were reviewed qualitatively (phase II) and quantitatively (phase III). The exclusion criteria for phase II were identical to those used in phase I in addition to: 1) high potential for information bias (defined as no neuroimaging (CT-scans or MRI) or autopsies used for the diagnosis of NCC); 2) high potential for selection bias (defined as the study of volunteers or less than 80% of patients with imaging and NCC); or 3) all available data were from before 1990 or after June 1, 2008. The quantitative data from documents included after phase II were extracted in phase III.

Articles reporting the proportion of epilepsy cases with lesions of NCC were excluded from the current study and reported in another article [5].

### Data extraction

Data on studies' characteristics, methodological quality and frequency of clinical manifestations and mortality were collected. Data extraction was conducted independently by at least two investigators. A third investigator checked a random sample of 10% of all of the entries. Discrepancies were resolved through discussion until a consensus was reached. The screening process (phase I) was performed in an Excel® spreadsheet (Microsoft Corp., Redmond, WA). Methodological factors (phase II) and frequency data (phase III) were recorded in standardized electronic forms of a data extraction tool which was developed in Access® (Microsoft Corp., Redmond, WA) specifically for this review (available from the authors on request). Authors of primary studies were contacted when the article being reviewed contained missing or unclear information on the study design or results.

### Data synthesis and analysis

Whenever two or more different studies described the same clinical manifestation, we conducted a meta-analysis and estimated the pooled proportion of the given clinical manifestation among people with NCC. For these analyses, studies reporting “seizures” and those reporting “epilepsy” were combined, as most reports did not discriminate between the two. The definition of epilepsy is the occurrence of at least two unprovoked seizures separated by at least 24 hours [6].

As there was great variability in the characteristics of the included documents, results were expressed as random-effects models using proportion with 95% confidence intervals (95% CI) [7]. The Freeman-Tukey double arcsine transformation was used for pooled estimates of proportion and corresponding 95% CI from the random-effects model [8]–[9]. The Cochran's Q test was used to assess homogeneity across studies and the I^2^ index was used to summarize the total variability in proportion due to between-study variation [10]. Random-effect models were used due to important heterogeneity between studies. A sensitivity analysis was conducted by estimating the pooled proportion after omitting one study at a time. The analysis was performed with the R META package (Version 0.8–2; Guido Schwarzer in R-META metagen function) from R statistical software (R Development Core Team, www.R-project.org). No study had considerable effect on the pooled estimate and results from the sensitivity analyses are not presented. A mixed-effects regression model was used to determine if the age group (children vs adults) significantly influenced the estimated percentage of seizures and epilepsy among people with NCC.

## Results

### Literature search

**Figure 1:**
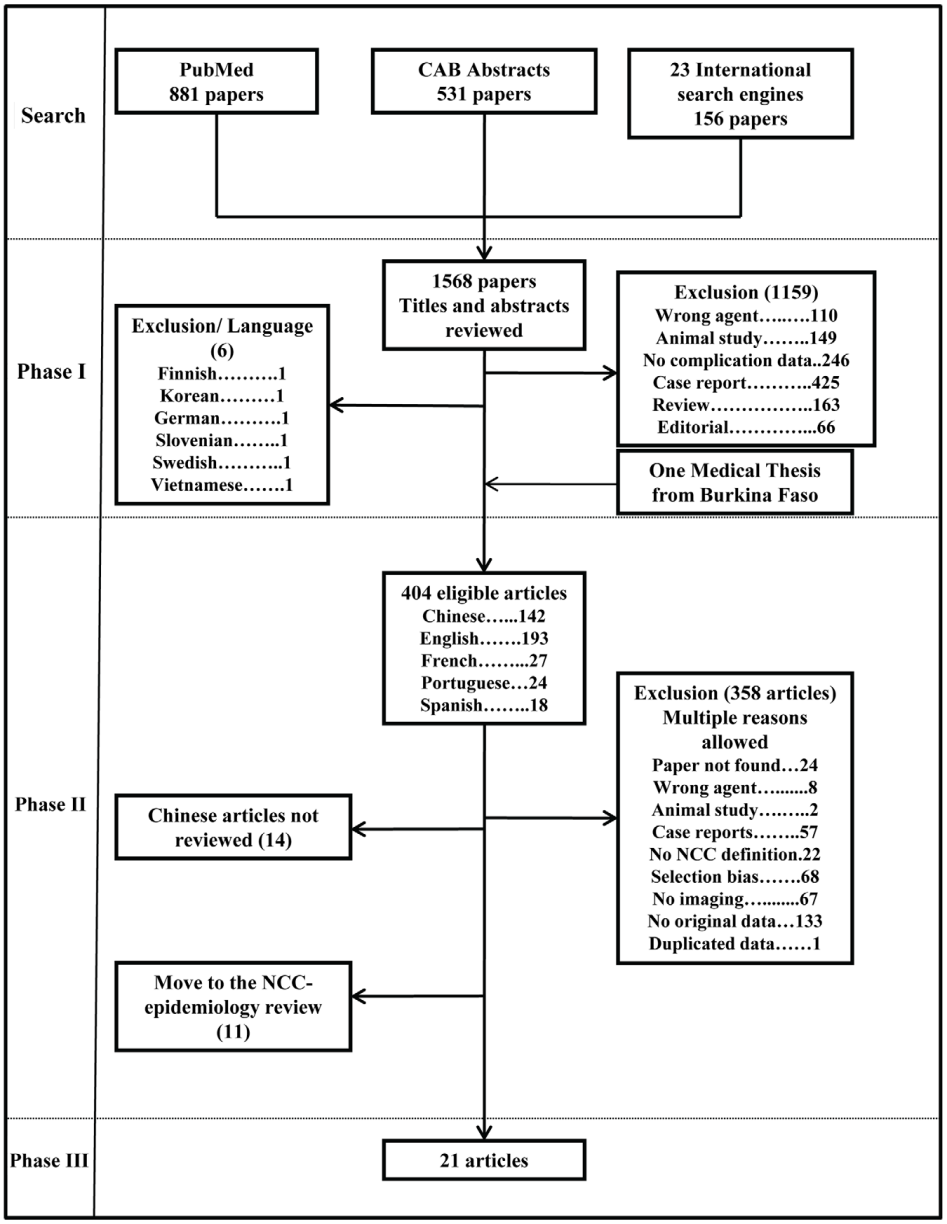
Flowchart describing the number of papers remaining at different phases of the study.

A total of 1569 documents were identified in phase I. Figure 1 shows the number of papers identified in each database and included in each phase and the reasons for exclusions. After phase I, nearly three-quarters of the articles were excluded. An additional 383 articles were excluded during phase II, most of which (n = 200) did not have manifestation data or did not use neuroimaging to diagnose NCC. Fourteen Chinese articles could not be traced and were excluded. Finally, 11 articles were excluded from this review and included in a study on the proportion of epilepsy cases with NCC (see [5]).

Phase III included 21 documents (1.3%) containing quantitative data on various clinical manifestations associated with NCC (Table 1).

**Table 1:** Descriptive summary of the studies included for estimating the distribution of manifestations associated with neurocysticercosis.

Reference (language)	Country, year(s) of study	Population			Diagnosis of NCC	Type of lesions	Measurement of manifestations
		Source	Target	Study sample size			
[16] (Portuguese)	Brazil, 1987–97	Autopsies of the Serviço de Anatomia Patológica do Hospital de Clínicas da Universidade Federal do Paraná, 1987–97	28 autopsies (>15 years old)	27 (>18–85 years old)	Autopsies	100% active	Medical chart and necropsy report
[15] (Portuguese)	Brazil, 1995–6	Catchment population of the Centro de Diagnostico por Imagen do Parana (CEDIP), Hospital das Nacoes, Curitiba, PR, 1995–96	236 neurology patients with NCC (all ages)	236	CT-scan[49]	7.2% active	Not specified
[11] (English)	Brazil, 1993–4	People attending the Section of Neuroinfectious Diseases at the Clinicas da Faculdade de Medicina da Universidade de Sao Paulo, 1993–94	38 neurology patients with NCC (18–60 years old)	38	CT-scan + positive CSF immunological test + MRI (cystic lesions)	76.3% active	Interview (depression) and medical charts
[25] (Portuguese)	Brazil, 1990–01	Catchment population of Ambulatorio de Neurologia do Hospital Universitario Alcides Caeneiro, Paraiba, 1990–01	44 neurology patients with NCC (all ages)	44	CT –scan[50] + CSF (33)+MRI (5)	52.3% active	Standardized medical chart reviews
[21] (English)	Ecuador, 1985–98	Catchment population of the Department of Neurology of the Eugenio Espejo Hospital in Quito, 1985–88	420 neurology patients with a stroke (17–86 years old)	420	CT scan, CSF, immunologic tests, other tests[51]	NR	Stroke defined according to Kotila, 1984 (neurological examination)
[32] (English)	Ecuador, 1994–96	Catchment population of the Department of Neurology, Luis Vernaza Hospital, and at the Neuro-Oncology Service, Instituto Oncologico Nacional, Guayaquil, 1994–96	43 neurology patients with cerebral glioma (20–86 years old)	43	CT-scan[52]–[53]	100% inactive	Histology of open biopsy (presence of malignant glial cells)
[20] (English)	Mexico, 1989–96	Catchment pediatric population of the Neurology Department of the Instituto Nacional de Pediatria in Mexico City, 1989–96	122 neurology patients with NCC (14 months to 17 years old)	122	CT-scan, CSF (n = 71), MRI (n = 20), immunological tests	82.0% active	Medical charts
[12] (English)	Mexico, 1993–03	Catchment population of three referral hospitals, Mexico City, 1993–03	206 neurology treatment-free NCC patients (11 months - 62 years old)	206	CT-scan and/or MRI	75.7% active	Direct questionnaire (adults, 1 year prospective) or hospital records (children, retrospective)
[33] (English)	Mexico, 1993–96	People autopsied at the General Hospital of Mexico, 1993–96	113 autopsies with malignant hematological diseases (0–80 years old)	113	Pathological analysis of the brain (autopsy)	NR	Not provided (autopsy)
[34] (Spanish)	Mexico, 1986–98	Catchment population of the neurological clinic of the Instituto Nacional y Neurologia Manual Velasco Suarez, 1986–98	63 patients with non-aneurysmal sub-arachnoid hemorraghe (19–82 years old)	50	CT-scan and MRI (no specific definition provided)	NA	Spontaneous headache or alteration of consciousness w/o trauma and presence of blood in the subarachnoid space
[22] (English)	USA, 1985–91	Catchment population of the Ben Taug General Hospital of Houston, Houston Texas, 1985–91	112 patients with NCC (1–84 years old)	112	Discharge diagnosis of NCC (definite or probable, no reference)	80.4% active	Medical charts
[23] (English)	USA, 1986–94	Catchment pediatric population of the at Children's Memorial Hospital's emergency room, Chicago, 1986–94	47 children with NCC (1–15 years old)	47	Biopsy or MRI/CT-scan and serological or CSF tests or MRI/CT and epidemiological link	56% active from 45 CT-scans	Medical records (medical, laboratory, pathology, outpatient records)
[18] (English)	India, 1984–87	Pediatric catchment population of the G.B. Pant Hospital, New Delhi, 1984–87	27 children with NCC (3–12 years old)	27	CT-scan and positiveCSF ELISA and MRI and histological exam	81.4% positive for CSF ELISA	Not specified
[19] (English)	India, 1979–90	Pediatric Neurology Clinic patients, New Delhi, 1979–90	50 neurology patients with NCC (1–15 years old)	50	CT-scan supported by MRI (8), history, serum or CSF antibodies, histology of nodules	80% active	Medical charts
[24] (Chinese)	PR China, date?	Hospitalised population of the department of neurology, Guangdong Medical University Hospital, Guangdong Province, no dates	36 inpatients with NCC (14–60 years old)	36	CT (31) or MRI (9) of the brain[54]	78% with active lesions	Not specified
[27] (Chinese)	PR China, 1995–01	Catchment population of the department of infectious disease, Huaghan Hospital, Shangai, 1995–01	125 patients with NCC (2–68 years old)	125	MRI or CT scan of the brain showing lesions of NCC (3 were normal)	90 active, 4 inactive, 31 unknown	Not specified
[26] (Chinese)	PR China, 1997–01	Catchment population of the NCC institute of the Jilin University, 1997–01	210 patients with NCC (< 15 years old)	210	MRI or CT scan of the brain AND immunological test positive in the CSF or serum	83.7% with active lesions	Medical chart reviews
[14] (English)	Portugal, 1983–92	Catchment population of the reference Hospital Geral de San Antonio, district of Oporto, 1983–92	38 patients referred for a CT active NCC (6–62 years old)	38	CT-scan (“true” or “probable”)[55]	100% active	Not specified
[13] (English)	Portugal, 1983–89	Catchment population of the reference Hospital Geral de San Antonio, district of Oporto, 1983–89	231 symptomatic patients referred for a CT with NCC (age unknown)	144 with symptoms	CT-scan (divided as “true” and “probable”)[55], MRI, CSF ELISA	20.0% active; 80.0% inactive	Not specified
[56] (French)	Burkina Faso, 2001–06	Catchment population of the neurology and radiodiagnosis of the Centre Universitaire Yaldago Ouédraogo, Ouagadougou, 2001–06	35 symptomatic patients referred for a CT with NCC (8–78 years old)	35	CT-scan	74.3% active, 25.7% inactive	Medical examination (prospective)
[17] (English)	South Africa, 1984–86	Catchment population of the Garankuwa hospital, 1984–86	88 patients with NCC (adults)	88	CT-scan	100% active	Not specified

NA: Not applicable.

NR: Not Reported.

### Literature search on death rate associated with NCC

A total of 45 publications were identified. Of those, 27 were excluded in phase I (seven case reports, 14 reviews or letters, two animal studies, four without death data). Eighteen studies were read in full. Of those, two were case reports, nine did not provide an estimate of death rate and three were reviews. Table 2 reports the four studies included.

**Table 2:** Descriptive summary of the studies included for estimating the death rate associated with neurocysticercosis.

Reference(language)	Country, year(s) of study	Denominator	Source of death data	Measure of mortality
[28] (English)	USA, 1990–02	Population of the United States 1990–02	National Center for Health Statistics (NCHS)	Age adjusted annual mortality rate
[29] (English)	Brazil, 1985–04	Population of the state of Sao Paolo, 1985–04	Death certificates	Over 20 years age- standardized mortality rates
[30] (English)	USA, 1989–00	Population of California 1989–00	State of California, Center for Health Statistics, Office of Vital Records	Crude 12 years mortality rate
[31] (English)	USA, 1995–00	Population of Oregon 1995–00	State of Oregon Death certificates	Crude 6 years mortality rate

### Distribution of manifestations among people with NCC attending neurology clinics

People diagnosed with NCC presented with a wide range of clinical manifestations. Definitions and measurement methods for manifestations were very rarely provided (Table 1).

**Figure 2:**
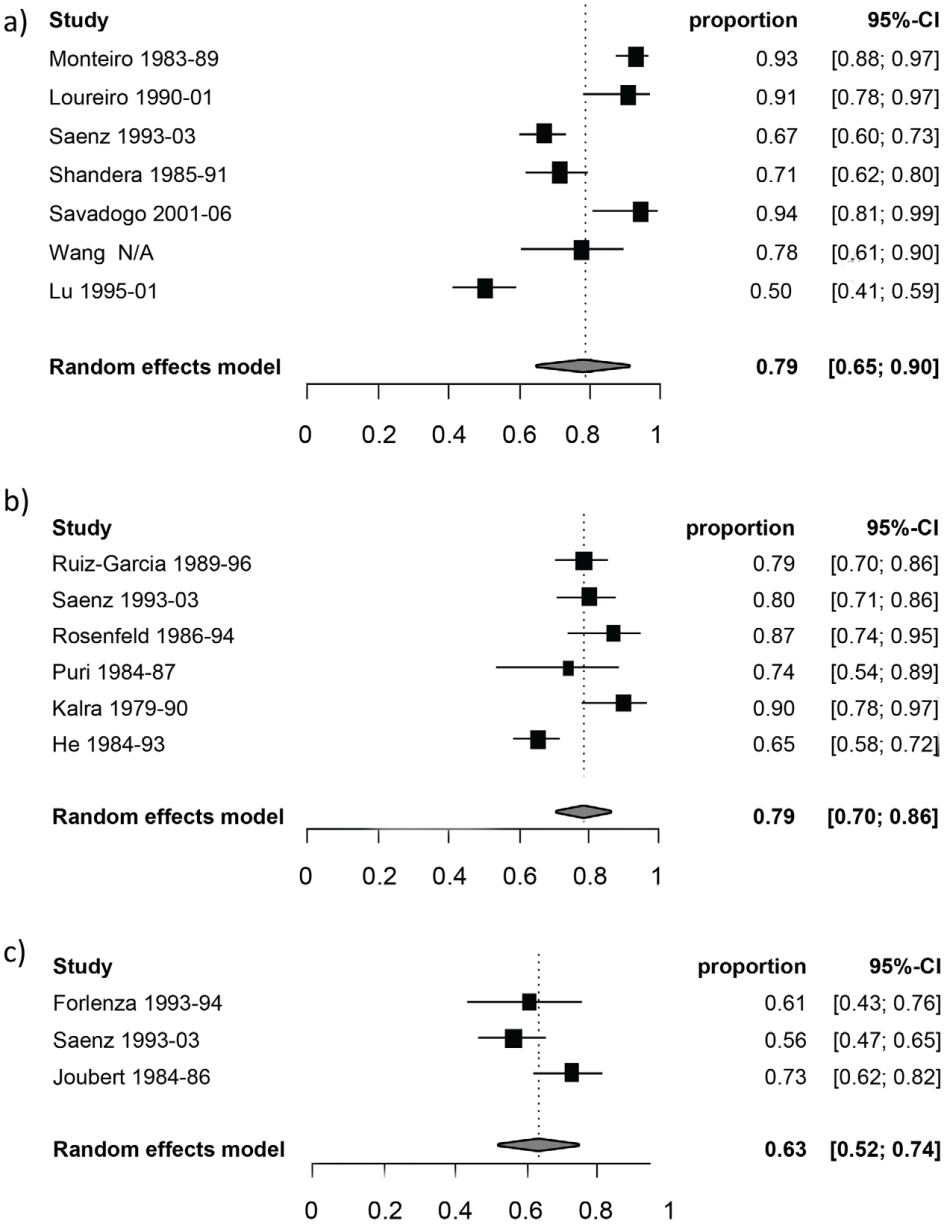
Forest plots of the proportion of symptomatic neurocysticercosis cases presenting with seizures/epilepsy. The forest plots represent A) all age groups, B) Children (0–19 years old) and C) Adults (>19 years old). N/A represents the period of study missing.

**Figure 3:**
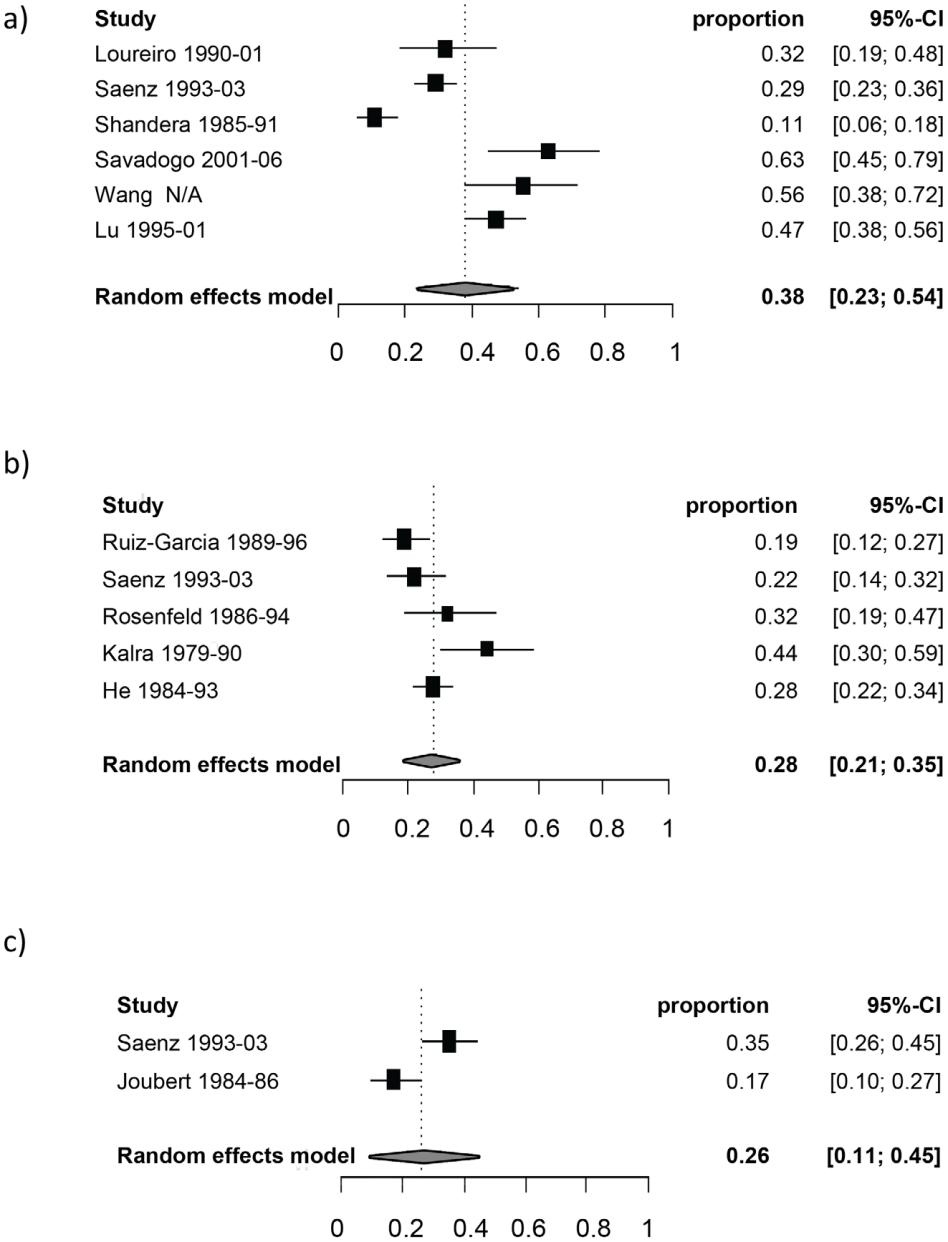
Forest plots of the proportion of symptomatic neurocysticercosis cases presenting with headaches. The forest plots represent A) all age groups, B) Children (0–19 years old) and C) Adults (>19 years old). N/A represents the period of study missing.

**Figure 4:**
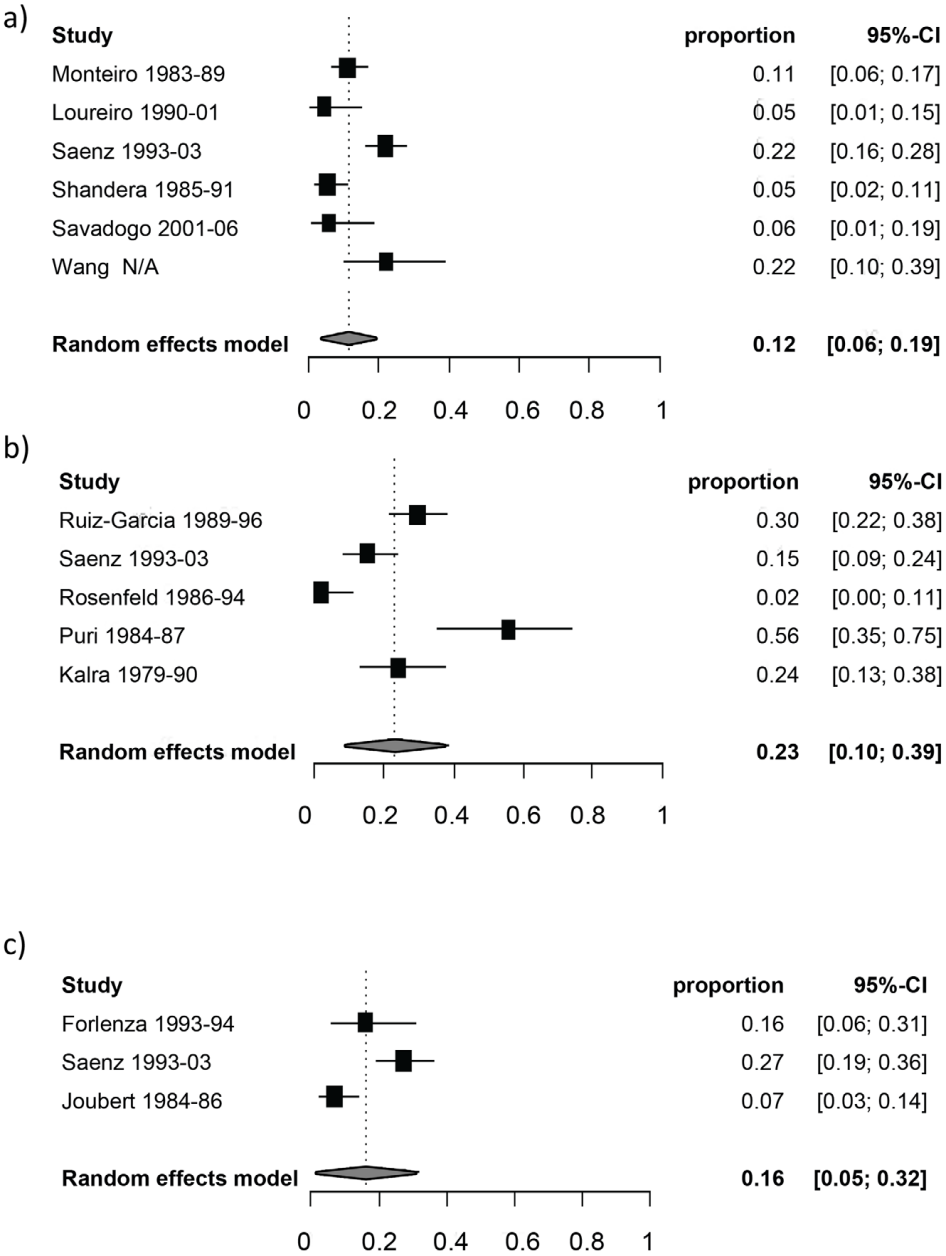
Forest plots of the proportion of symptomatic neurocysticercosis cases presenting with increased intracranial pressure symptoms. The forest plots represent A) all age groups, B) Children (0–19 years old) and C) Adults (>19 years old). N/A represents the period of study missing.

**Figure 5:**
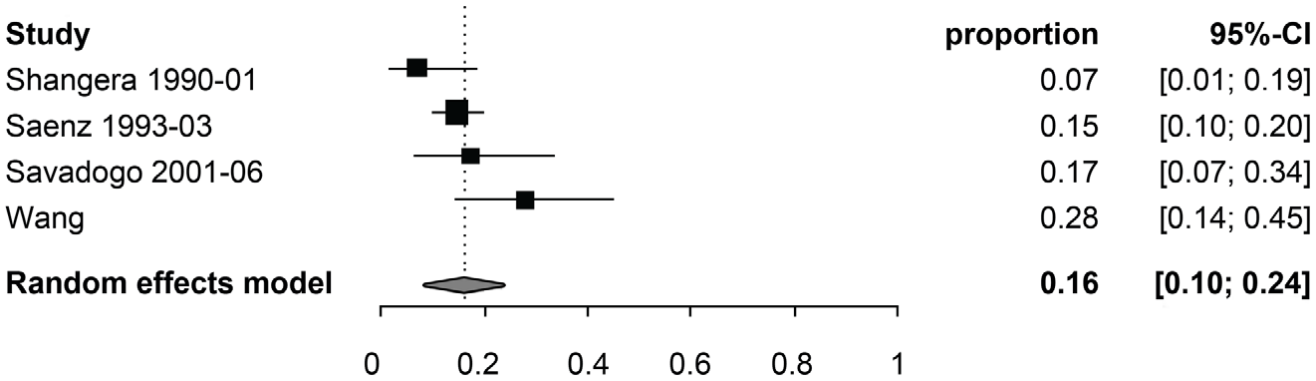
Forest plots of the proportion of symptomatic neurocysticercosis cases presenting with focal deficits. N/A represents the period of study missing.

**Figure 6:**
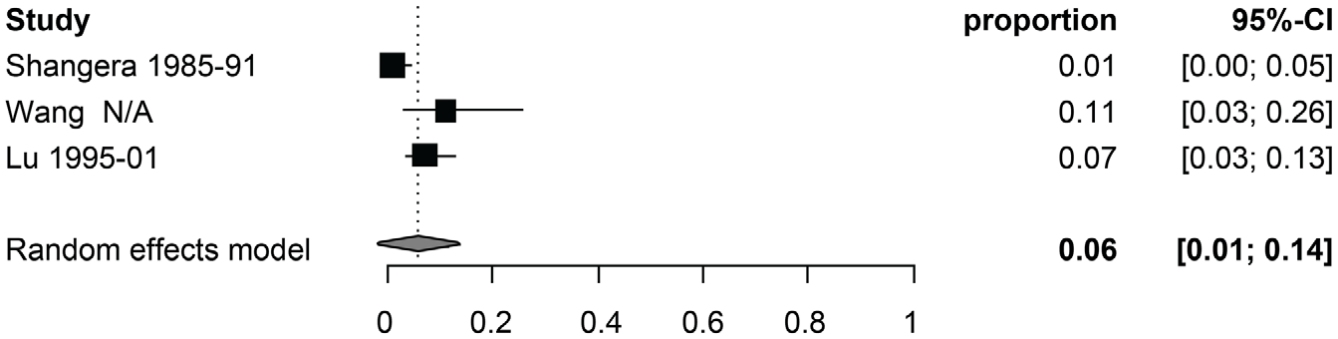
Forest plots of the proportion of symptomatic neurocysticercosis cases presenting with visual changes. N/A represents the period of study missing.

**Figure 7:**
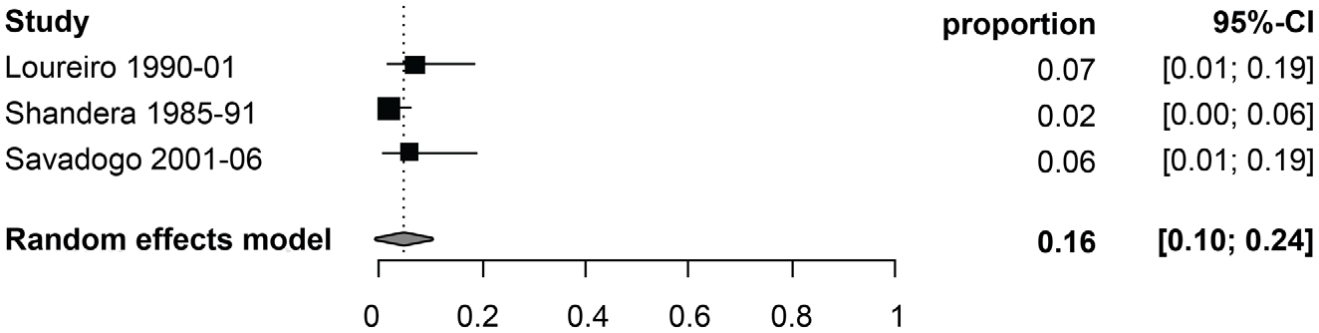
Forest plots of the proportion of symptomatic neurocysticercosis cases presenting with altered mental state.

Table 3 reports the random-effect model pooled estimates (where applicable) of the percentage of manifestations among symptomatic NCC patients by age group. Figures 2 and 3 report the corresponding forest plots for epilepsy and headaches. Forest plots for the other manifestations are shown in Figures 4, 5, 6, 7. Each forest plot illustrates the estimated percentage and 95%CI of NCC patients with the manifestation of interest found in each study. The losange at the bottom of each plot corresponds to the estimated pooled estimate based on the random-effects model. In all age groups, seizures/epilepsy was by far the most common manifestation followed by headaches, focal deficits and signs of increased intracranial pressure. All other manifestations were estimated to occur in less than 10% of symptomatic NCC patients presenting for care. The estimated proportion of seizures/epilepsy among children NCC patients was 15.9% (95%CI: 2.3%–29.5%) higher than in adult NCC patients. All other manifestations, except for altered mental state, occurred in a similar proportion of pediatric and adult NCC patients. One study focusing on psychiatric symptoms among NCC patients [11] was an outlier, which explains the very large 95% confidence interval for this estimate among adults.

**Table 3:** Pooled estimates of the percentage of manifestations among symptomatic NCC patients using random-effect binomial models.

Manifestation		Age group	
	All	Children	Adults
Seizures/epilepsy	78.8% (65.1%; 89.7%)	78.9% (70.5%; 86.2%)	63.2% (51.9%; 73.8%)
Headaches	37.9% (23.3%; 53.7%)	27.7% (20.7%; 35.2%)	25.9% (10.7%; 45.0%)
Signs of Intracranial Pressure/Hydrocephalus/Papilledema	11.7% (6.0%; 18.9%)	22.7% (10.2%; 38.5%)	16.3% (5.3%; 31.8%)
Meningitis symptoms	7.9% (2.7%; 15.5%)	11.2% (5.2%; 19.0%)	5.6% (1.9%; 12.8%)*
Cranial nerve palsy	2.8% (0.1%; 14.5%)*	6.0% (0.6%; 16.2%)	NA
Gait abnormality/ataxia	6.0% (1.9%; 12.1%)	2.4% (0.2%; 7.2%)	5.6% (1.9%; 12.8%)*
Focal deficits	16.0% (9.7%; 23.6%)	12.5% (7.6%; 18.4%)	11.8% (4.1%; 22.9%)
Visual changes	5.6% (1.1%; 13.5%)	3.5% (1.3%; 6.7%)	NA
Altered mental state/psychiatric symptoms	4.5% (1.5%; 9.0%)	4.0% (0.5%; 13.4%)*	28.1% (0.5%; 74.9%)
Pyramidal signs	NA	11.6% (0.0%; 42.9%)	NA

*: One study with binomial 95%CI.

NA: No data Available.

The distribution of manifestations in patients with active and inactive lesions was presented in three studies [12]–[14] (Table 4). There was a considerably higher proportion of patients with inactive NCC who presented with seizures/epilepsy (>88%) as compared with patients with active lesions (60–63%). Conversely, the proportion with signs or symptoms of intracranial hypertension was approximately 25% in patients with active lesions but was not found in patients with inactive NCC lesions. Hydrocephalus and meningitis were reported by only one author and were more frequent with active lesions.

**Table 4:** Percentage of manifestations reported in symptomatic NCC patients with active and inactive lesions.

Reference	Country, year(s) of study	Manifestation	Type of lesions	Percentage	95%CI
[14]	Portugal, 1983–92	Seizures/Epilepsy	Active	60.5%	43.4%–76.0%
[12]	Mexico, 1993–03	Seizures/Epilepsy	Active	62.8%	54.7%–70.4%
[13]	Portugal, 1983–89	Seizures/Epilepsy	Inactive	98.3%	92.6%–99.5%
[12]	Mexico, 1993–03	Seizures/Epilepsy	Inactive	88.0%	75.7%–95.5%
[14]	Portugal 1983–92	Intracranial Hypertension	Active	23.7%	11.1%–40.2%
[12]	Mexico, 1993–03	Intracranial Hypertension	Active	28.8%	21.9%–36.6%
[13]	Portugal, 1983–89	Intracranial Hypertension	Inactive	NA	NA
[12]	Mexico, 1993–03	Intracranial Hypertension	Inactive	0.0%	0.0%–7.1%
[14]	Portugal, 1983–92	Hydrocephalus at CT	Active	23.7%	11.4%–40.2%
[13]	Portugal, 1983–89	Hydrocephalus at CT	Inactive	3.5%	1.0%–8.7%
[14]	Portugal, 1983–92	Meningitis	Active	5.3%	1.7%–21.4%
[13]	Portugal, 1983–89	Meningitis	Inactive	0.9%	0.0%–4.7%

### Distribution of manifestations in people with NCC attending an imaging clinic

In one study from Brazil [15], 236 patients seen at an imaging clinic had lesions suggestive of NCC of which 219 were inactive lesions. Lesions suggestive of other pathologies were found in 48 (20.3%) of the cases with suspected NCC. The distribution of manifestations was 30% with epilepsy, 51% with headaches, 8% with focal motor/sensory deficits. There were 35% with “other” symptoms found among patients with a CT-scan due to suspected neoplasia or stroke.

Among 231 patients with NCC lesions seen in a neuroradiology department, 87 (38%) were asymptomatic, incidental findings in trauma patients and cases of suspected cerebrovascular disease [13]. All of these 87 had inactive lesions. This study is unique because it reports on the possible clinical spectrum of NCC.

### Distribution of manifestations among autopsied patients

In a review of 901 autopsies conducted in a department of pathology and anatomy at the University Hospital of Paraná, Brazil, the authors reported on 28 cases of NCC, with medical charts available for 27 of the cases [16]. Of those, 13 were asymptomatic, nine had seizures as a complicating factor of the clinical picture prior to death, four had increased intracranial pressure, one had meningitis, one had a cerebrovascular form and one had dementia noted at some point during the course of the disease. Only two of the 27 patients had been diagnosed with NCC prior to death, which suggests that NCC is often undiagnosed among patients with neurological symptoms.

### Proportion of NCC cases seeking care who died

The proportion of cases of NCC who sought care and subsequently died were 2.3% (2/88) of adult patients with active lesions in South Africa [17], 18.5% (5/27), 2.0% (1/50) and 1.6% (2/122) of pediatric patients in India [18]–[19] and Mexico [20], respectively, 3.2% (1/31) of adult patients with stroke in Ecuador [21], 5.3% (2/38) of patients with active lesions in Portugal [14], and 0.9% (1/112) of patients in Houston, Texas [22]. Most deaths were associated with complications of shunt surgery for the treatment of hydrocephalus. The duration of patient follow-up and referrals to other facilities were not reported, which limits the interpretation of the data. In a study of 27 autopsied patients in Brazil, the NCC lesions were considered the cause of death in 30% of the autopsied cases [16].

### Duration of disease at the time of seeking medical care

Several of the studies reported the time from the onset of symptoms to seeking medical care, with an average of 56.8 weeks in adult patients in South Africa [17], a median of 3.5 months (range 0–492 months) in patients in the United States [22], a median of 2 days (range <1 day to 8.75 years) in children presenting to the emergency room in the United States [23], and a range of 5 days to 20 years in inpatients seeking care in China [24]. Percentages of 77.3% [25] and 80% [26] of patients had sought care within one year, 92.8% within three years [27]. The time from onset of symptoms to seeking and receiving medical care will also vary depending on the type of manifestations and the local medical services' capacity.

### Death rate due to NCC

One study in the United States [28] and another in the State of Sao Paolo [29], Brazil, reported age-adjusted annual mortality rates of 0.06 (95% CI: 0.05–0.07) and 1.68 (95% CI: 1.58–1.78) deaths per million population, respectively. The other studies from California [30] and Oregon [31] in the United States reported annual crude mortality rates of 0.33 (95% CI: 0.27 – 0.38) and 0.29 (95%CI: 0.11–0.64) deaths per million population.

### Proportion of NCC cases among people with specific manifestations

In people presenting with glioma [32], malignant hematological disease [33], or non-aneurysmal subarachnoid hemorrhage [34], the proportions with NCC were 18.6%, 6.2% and 4.0%, respectively (Table 5). The proportion of NCC reported in people with stroke was 7.4% [21]. Some correlation between the location of the cyst and the focal neurological deficit was found in all NCC cases. These studies can only be used to encourage physicians to add NCC to their list of differential diagnoses when such a manifestation occurs, especially in endemic countries.

**Table 5:** Percentage of NCC among people presenting in specific populations.

Reference	Country, year	Clinical presentation	Number of people with NCC	Number of people with manifestations	% NCC (95% CI)	
[32]	Ecuador, 1994–96	Cerebral Glioma	8	43	18.6%	7.0%–30.2%
[33]	Mexico, 1993–96	Malignant hematological disease	7	113	6.2%	1.8%–10.6%
[19]	Ecuador 1985–88	Stroke	31	420	7.4%	5.1%–10.3%
[34]	Mexico 1986–98	Non-aneurysmal subarachnoid hemorrhage	2	50	4.0%	0.1%–11.6%

### Association between manifestation and NCC

The odds ratio of NCC and cerebral glioma was estimated to be 7.63 (95%CI: 2.03–31.09) when cases of glioma were compared to age-sex-socioeconomic status matched, previously healthy, head trauma controls [32]. The odds ratio of the relation between NCC and malignant hematological diseases was estimated to be 3.54 (95%CI: 1.17–9.79) when autopsied cases were compared to autopsied cases without any type of neoplasm [33].

## Discussion

This study is the first systematic review of clinical manifestations associated with NCC, which can have a wide spectrum of neurologic and psychiatric manifestations including seizures, epilepsy, headache, cerebrovascular disorders, motor deficits and depression [35].

More than three-quarters of symptomatic NCC patients seen in neurological clinics present with seizures or epilepsy. Although definitions of these conditions were very rarely provided, the estimate was surprisingly consistent across studies as a result of including only studies of a certain quality, making them more comparable to one another and the results more valid. Several recent review papers have reported percentages of NCC cases presenting with seizures and epilepsy varying from 70% to 90% [36]–[42].

The proportion of NCC cases seen in neurological clinics with seizures/epilepsy was higher in children than adults. In a review paper of NCC in childhood from India, the authors reported that from 70% to 90% of children with NCC present with seizures [43], which agrees very well with our finding. However, these results may also reflect the fact that more children with seizures/epilepsy are referred to facilities with CT as compared to adults. In addition, if adults tend to be referred to neurology clinics for a larger spectrum of neurological disorders, this would reduce the proportion of seizure/epilepsy observed.

The next most common manifestation was headaches, at a frequency of approximately one-third of symptomatic NCC patients. The between-study estimates were more variable than what was seen for seizures/epilepsy, but were still reasonably consistent. This is surprising, since no study provided a definition for headaches. The proportion of pediatric patients with headaches was similar to that in adults but lower than the estimate for all ages combined. Measuring headaches in toddlers and young children is especially challenging since most of them cannot communicate their symptoms [44].

The effect of NCC on altered mental state and psychiatric symptoms remains poorly described. However, in the studies that were included here, they were the presenting manifestations in about 5% of cases of NCC, except for one study [11], where 52% were found to have depression at presentation. Had the studies also included psychiatry clinics, these estimates may have been higher. The proportion of NCC cases with symptoms of or increased intracranial pressure was similar between children and adults. This could be due to the fact that papilledema, which is more common among children, was included in this category of symptoms.

All of the publications found in this review reported on patients with symptomatic NCC seen in neurology clinics where imaging was available. Therefore, the distribution of manifestations over-estimates the true frequency of NCC-associated disease, since patients who are asymptomatic or with only mild symptoms are unlikely to be seen in neurology clinics. Indeed, in two studies which were conducted in neuroimaging departments, about 35% of all NCC cases were asymptomatic [14]–[15]. In an autopsy study, nearly 50% of cases of NCC did not have symptoms noted in their medical charts [16]. There is a lack of knowledge on the proportion of NCC cases who will develop symptoms, when in the course of disease specific symptoms occur, and the frequency with which the manifestations change over time. In a study conducted in Mexico, 9.1% of randomly-selected residents without neurological symptoms were found to have NCC based on CT-scan examinations [45]. In another study conducted in Honduras with sampling based on EITB results, 31 of 148 participants (21%) had lesions of NCC. Of these, 26 (18%) showed no manifestations, two had headaches, two had epilepsy, and one had dizziness [46]. The authors demonstrated that EITB had very poor accuracy in detecting NCC, which would suggest that sampling based on EITB may not introduce any important selection bias. If this is the case, then we could conclude that 16.1% (5/31) of people in that community had prevalent NCC symptoms. Unfortunately, in none of those studies was the history of manifestations reported.

Assessing the distribution of manifestations among people with active and inactive lesions can inform us somewhat about the natural history of NCC. Seizures and epilepsy were more frequent among patients with calcified lesions. Those with active lesions were more likely to present with increased intracranial pressure, hydrocephalus or meningitis. If properly defined, the term “epilepsy” would be used to include only persons with unprovoked, recurrent seizures [6]. Thus, any cases of epilepsy that were a result of NCC would, by definition, have to occur in persons with inactive lesions, otherwise they would be acute symptomatic seizures. The higher proportion of seizures/epilepsy in those with inactive lesions may also reflect that NCC and epilepsy may be co-occurring conditions rather than be causally linked.

The duration of NCC-associated disease remains unknown. This review of the literature only allowed the estimation of the time between the first recorded or reported symptom and medical care. Some patients will never seek care and the duration of disease will remain unknown since NCC can only be accurately diagnosed with imaging. Once patients are in care, in case of active disease, cysticercosis will be treated and the symptoms will most likely disappear, although in the case of seizures, they may persist beyond the period of active disease.

Death was reported in only a few studies. It has been reported that neurologic deterioration in patients with NCC may be a life-threatening event with numerous causes and diverse clinical presentations [40]. In one study, the principal concurrent conditions listed as contributing to death included hydrocephalus, cerebral edema, cerebral compression, and epilepsy/convulsions [28]. The methods to estimate death rates were so heterogeneous that they could not be combined. In order to estimate the global burden of NCC, using a country-specific case fatality rate would be more helpful.

Various uncommon clinical manifestations have been reported in numerous case reports, illustrating that clinical manifestations associated with NCC are non-specific and pleomorphic [35], [40]. However, in this review, only case series that had more than 20 participants were included meaning that rarer manifestations are not included. Another important limitation is the lack of definitions of the outcomes of interest. In addition, it is possible that some researchers chose to report on only a certain set of symptoms and not on others.

Alertness of medical staff is needed to better recognize and diagnose NCC, including providing symptoms' definitions to improve our knowledge of its clinical spectrum. Other limitations are inherent to NCC itself and result from the difficulty of diagnosing NCC even with neuro-imaging [47]. Brain calcifications or granulomas which represent the most frequently observed feature in NCC are also common in tuberculosis, sarcoidosis and toxoplasmosis [48]. These lesions may lead to false positive NCC diagnoses and biased estimates of symptoms' distribution [46].

This systematic review of the literature shows that NCC imposes a heavy burden in endemic communities causing a wide range of neurological, neuropsychological and psychiatric manifestations and even premature death. Some clinical manifestations have an insidious onset and a slow progression, making their diagnosis difficult and often delayed. Hence, NCC should be kept in mind when confronted with any neurological manifestation in patients with histories of residing in endemic areas. When the clinical presentation suggests NCC infection, it is critical to perform a neuroimaging examination. The development of modern, affordable, valid diagnostic procedures and tests is needed to improve understanding of all the clinical manifestations of NCC and its epidemiology. A highly sensitive, specific and inexpensive diagnostic tool will represent a big step in gaining insight into the morbidity and mortality caused by NCC and will help to accurately estimate its global burden.
